# Development and Characterization of Sustainable Epoxy Biocomposites Reinforced with Coconut Shell Powder and GNP

**DOI:** 10.3390/polym18141728

**Published:** 2026-07-14

**Authors:** Muhammet Aydın, Maruf Hurşit Demirel, Ercan Aydoğmuş

**Affiliations:** 1 Department of Mechatronics Engineering, Faculty of Engineering, Fırat University, Elazığ 23119, Türkiye; muhammeta@firat.edu.tr; 2 Department of Basic Sciences, Faculty of Pharmacy, Fırat University, Elazığ 23119, Türkiye; mhdemirel@firat.edu.tr; 3 Department of Chemical Engineering, Faculty of Engineering, Fırat University, Elazığ 23119, Türkiye

**Keywords:** epoxy-based biocomposites, coconut shell powder, graphene nanopowder, thermal and dielectric properties, mechanical and microstructural characteristics

## Abstract

The development of sustainable polymer composites reinforced with renewable resources and advanced nanomaterials has attracted considerable attention for multifunctional engineering applications. In this study, an environmentally friendly epoxy-based biocomposite (EBC) reinforced with coconut shell powder (CSP) and graphene nanopowder (GNP) was successfully produced through a casting process. CSP was employed as a bio-based filler, while GNP was incorporated at concentrations ranging from 0 to 0.75 wt.% to improve the overall performance of the composites. The effects of GNP loading on bulk density, tensile strength, elongation at break, Shore D hardness, thermal conductivity, dielectric properties, thermal stability, mechanical and microstructural characteristics were systematically investigated. The results demonstrated that the incorporation of GNP significantly enhanced the multifunctional properties of the improved EBCs. Bulk density increased from 1137.5 to 1143.1 kg m^−3^ with increasing GNP content. The optimum tensile strength of 28.6 MPa and Shore D hardness of 77.4 were achieved at 0.45 wt.% GNP, indicating effective stress transfer and strong interfacial interactions between the epoxy matrix, CSP, and GNP. Thermal conductivity increased from 0.110 to 0.149 W m^−1^ K^−1^, while the dielectric constant increased from 3.06 to 4.25 with increasing GNP concentration. Thermogravimetric analysis revealed improved thermal stability and enhanced char formation in graphene-containing composites. FTIR analysis confirmed that graphene acted primarily as a physical reinforcement without altering the fundamental chemical structure of the epoxy network. SEM and EDX investigations demonstrated improved structural compactness, homogeneous filler dispersion, and successful graphene incorporation. The findings indicate that GNP and CSP reinforced EBCs possess significant potential for lightweight structural materials, thermal management systems, dielectric components, and sustainable multifunctional engineering applications.

## 1. Introduction

Polymeric composite materials have attracted significant attention in recent decades owing to their outstanding combination of lightweight structure, high specific strength, corrosion resistance, ease of processing, and design flexibility [1]. Among various polymer matrices, epoxy resins are widely utilized in aerospace, automotive, marine, electronic, construction, and energy-related applications due to their excellent mechanical performance, dimensional stability, chemical resistance, and strong adhesion characteristics [2]. Nevertheless, conventional epoxy systems are primarily derived from petroleum-based resources and often exhibit intrinsic brittleness, limited crack resistance, and relatively low sustainability, which have encouraged researchers to develop environmentally friendly and multifunctional composite systems [3]. 

The increasing environmental concerns associated with industrialization and the depletion of fossil resources have accelerated the search for sustainable materials derived from renewable and waste-based feedstocks [4]. Agricultural residues and agro-industrial wastes have emerged as promising reinforcement materials for polymer composites because they are abundant, inexpensive, biodegradable, and environmentally benign [5]. The utilization of agricultural by-products as fillers not only reduces material costs but also contributes to waste management and supports circular economy strategies by converting low-value residues into high-performance engineering materials [6].

In recent years, numerous lignocellulosic materials, including rice husk, wheat straw, corn stalk, coconut fiber, wood flour, sugarcane bagasse, and various nutshell wastes, have been incorporated into thermoplastic and thermosetting polymers [7]. These natural fillers contain cellulose, hemicellulose, and lignin, which provide relatively good stiffness and reinforcement capability [8]. Furthermore, the rough surface morphology and porous structure of many agricultural wastes may enhance mechanical interlocking with polymer matrices [9]. However, the incorporation of natural fillers can also introduce challenges such as increased moisture absorption, reduced interfacial compatibility, and heterogeneous dispersion within the matrix, which may adversely affect composite performance [10]. 

Among various agricultural residues, organic wastes have attracted increasing attention due to their high lignin content, remarkable hardness, excellent abrasion resistance, and significant carbonaceous structure. Large quantities of coconut shell residues are generated annually by food processing and agricultural industries, and a substantial portion of this biomass is discarded without value-added utilization. The transformation of coconut shell waste into reinforcing fillers for polymer composites represents a sustainable approach that can simultaneously reduce environmental burden and improve material performance. Previous studies have demonstrated that organic-derived fillers can contribute to improved stiffness, hardness, dimensional stability, and thermal resistance of polymeric materials depending on filler content, particle size, and processing conditions [11,12,13,14].

Although natural fillers can improve certain properties of polymer composites, their reinforcing efficiency is often limited by poor interfacial adhesion and relatively low intrinsic strength compared with advanced synthetic reinforcements [15]. Hybrid composite strategies combining bio-based fillers with nanoscale reinforcing materials have attracted considerable research interest [16]. Nanomaterials possess exceptionally high surface area-to-volume ratios and can significantly modify the mechanical, thermal, electrical, and barrier properties of polymers even at low loading levels [17]. The synergistic interaction between natural fillers and nanomaterials offers a promising route for developing multifunctional composite systems with enhanced performance [18].

GNPs are among the most attractive nanocarbon materials for advanced composite applications due to their extraordinary mechanical strength, high aspect ratio, excellent thermal conductivity, superior electrical conductivity, and large specific surface area [19]. Owing to these unique characteristics, graphene-based nanomaterials have been widely employed to improve the stiffness, fracture resistance, thermal stability, electromagnetic shielding capability, and dielectric behavior of polymer matrices [20]. Furthermore, GNPs are generally considered more economically viable than single-layer graphene while still retaining many desirable structural and functional properties. The incorporation of GNPs into epoxy systems has therefore become an active research area in both academic and industrial sectors [21,22,23].

Despite the extensive research conducted on graphene-reinforced epoxy nanocomposites and natural filler-based polymer composites, studies investigating the simultaneous incorporation of coconut shell waste and GNPs into epoxy matrices remain relatively limited [24,25]. In particular, the combined influence of bio-based coconut shell particles and nanoscale graphene reinforcements on the structural, mechanical, thermal, and dielectric performance of epoxy composites has not been comprehensively understood [26,27]. The interaction mechanisms among the epoxy matrix, lignocellulosic coconut shell particles, and GNPs may produce synergistic effects that cannot be predicted from single-filler systems [28,29,30]. Therefore, further investigation is necessary to clarify the role of graphene concentration in hybrid bio-nanocomposite structures.

In this study, environmentally friendly EBCs reinforced with CSPs and GNPs were successfully fabricated through a casting process. CSP was utilized as a sustainable biofiller, while GNPs were incorporated as multifunctional nano-reinforcements at different concentrations. The effects of graphene addition on the physical, mechanical, thermal, dielectric, and morphological characteristics of the resulting composites were systematically evaluated. The findings of this work are expected to contribute to the development of sustainable high-performance polymer composites by combining agricultural waste valorization with advanced nanomaterial technology, thereby providing new opportunities for engineering applications requiring lightweight, durable, and multifunctional materials.

## 2. Materials and Methods

### 2.1. Materials

A commercial two-component, solvent-free bisphenol-A-based epoxy resin system (Polisan Kansai Boya San. ve Tic. A.Ş., Kocaeli, Türkiye) was employed as the polymer matrix in the preparation of the epoxy biocomposites. The epoxy resin was cured using its corresponding polyamide hardener at the manufacturer’s recommended mixing ratio of 2:1 (*w*/*w*) (resin:hardener). According to the manufacturer’s technical specifications, the epoxy resin possesses a density of 1.20–1.24 g cm^−3^, a gel time of approximately 40–45 min at 23 °C, and achieves full curing and optimum chemical resistance after 7 days under ambient conditions. The cured epoxy system exhibits excellent adhesion to various substrates, good electrical insulation properties, and high resistance to water, chemicals, bacteria, and fungi, making it a suitable matrix material for high-performance structural and multifunctional composite applications.

CSP, an abundant agricultural by-product, was selected as the primary bio-based reinforcement because of its high lignocellulosic content, excellent hardness, low density, renewability, and environmental sustainability. Fresh coconut shells were collected from local food processing markets. The shells were manually cleaned to remove residual coconut flesh and impurities before being washed thoroughly with distilled water. The cleaned shells were oven-dried at 45 °C for 48 h until a constant weight was achieved. The dried shells were subsequently crushed using a laboratory crusher and ground in a high-speed mechanical grinder. The obtained powder was sieved through a standard sieve set, and particles smaller than approximately 149 μm were selected for composite fabrication to ensure homogeneous dispersion within the epoxy matrix.

GNPs were supplied by Nanografi Nano Technology (Ankara, Türkiye) and used as the nanofiller without further purification. According to the manufacturer’s technical data sheet, the GNPs possess a purity of 99.9%, an average platelet thickness of approximately 3 nm, an average lateral particle size of approximately 1.5 μm, and a specific surface area (BET) of approximately 800 m^2^ g^−1^. The material has a CAS number of 7782-42-5 and consists of graphitic carbon with a negligible degree of oxidation, making it suitable for high-performance polymer nanocomposites. The high specific surface area and nanoscale thickness of the graphene nanoplatelets promote effective stress transfer, enhanced interfacial interactions, and improved multifunctional properties in the developed epoxy biocomposites.

Triethylenediamine (TEDA) was used as a curing accelerator to improve curing kinetics and promote the formation of a highly crosslinked epoxy network. Analytical-grade acetone and ethanol were employed for cleaning laboratory equipment and sample preparation. Distilled water was used throughout the experimental procedure.

### 2.2. Preparation of CSP

The preparation of CSP consisted of sequential cleaning, drying, grinding, and particle classification processes. Initially, coconut shells were washed several times with distilled water to remove dust, oil residues, and surface contaminants. The cleaned shells were dried in a forced-air convection oven at 45 ± 1 °C for 48 h until constant mass was reached. After drying, the shells were mechanically crushed into coarse particles and subsequently pulverized using a laboratory mill operating at high rotational speed. The powder was sieved using stainless-steel laboratory sieves, and particles below 77 μm were collected for composite production. The fine CSP was stored in airtight desiccators at room temperature to prevent moisture absorption before composite fabrication.

### 2.3. Production of EBCs

Five different bio-based EBC formulations were prepared by varying only the GNP content while maintaining constant CSP and TEDA contents. The formulation compositions are presented in Table 1. Initially, epoxy resin (A) was weighed using an analytical balance with an accuracy of ±0.0001 g. The predetermined amount of GNP was gradually introduced into the epoxy resin and mechanically mixed at approximately 900 rpm for 15 min. To achieve homogeneous nanoparticle dispersion and minimize agglomeration, the mixture was further sonicated in an ultrasonic bath operating at 40 kHz for 30 min while maintaining the temperature below 35 °C. Following graphene dispersion, the pre-dried CSP was slowly incorporated into the graphene/epoxy suspension under continuous mechanical stirring for an additional 10 min. Subsequently, the required amount of TEDA accelerator was added and mixed for 5 min to ensure uniform distribution. Epoxy hardener (B) was introduced into the mixture according to the prescribed mixing ratio. The complete mixture was gently stirred for approximately 5 min to avoid excessive air entrapment while ensuring complete homogenization [31,32,33].

The resulting suspension was immediately poured into silicone molds previously coated with a commercial release agent. Degassing was carried out under vacuum for approximately 15 min to eliminate entrapped air bubbles and minimize internal porosity. The specimens were initially cured at ambient laboratory conditions (25 ± 2 °C and 50 ± 5% relative humidity) for 24 h, followed by post-curing at 50 °C for 4 h in a laboratory oven to complete the crosslinking reaction. After curing, all specimens were conditioned for at least 48 h at standard laboratory conditions prior to characterization.

**Figure 1:**
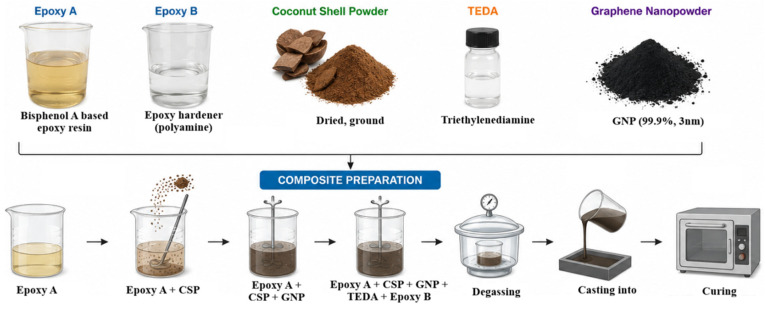
Schematic illustration of the experimental production process for EBC.

Figure 1 schematically illustrates the fabrication procedure of the EBCs reinforced with CSPs and GNPs. Initially, the required amount of dried and ground CSP was gradually incorporated into the bisphenol-A-based epoxy resin (Epoxy A) under continuous mechanical stirring to ensure homogeneous dispersion of the bio-based filler within the polymer matrix. Subsequently, the predetermined amount of GNP was added to the epoxy/CSP mixture and mixed thoroughly to promote uniform nanoparticle distribution and enhance interfacial interactions. After achieving a homogeneous suspension, the polyamine curing agent (Epoxy B) together with triethylenediamine (TEDA) accelerator was introduced into the mixture, followed by additional stirring to ensure complete blending of all constituents. The resulting composite slurry was then subjected to vacuum degassing to eliminate entrapped air bubbles generated during the mixing process, thereby minimizing internal void formation and improving the structural integrity of the composites. Finally, the degassed mixture was poured into the desired molds and cured under controlled conditions to obtain fully cross-linked epoxy biocomposites.

### 2.4. Bulk Density Measurement

The densities of the fabricated biocomposites were determined according to ASTM D792 [34] using Archimedes principle. Composite specimens were weighed in air and subsequently immersed in distilled water. Density values were calculated using the measured mass differences and water density. At least three specimens from each formulation were tested, and average values were reported [35,36].

### 2.5. Shore D Hardness Test

Surface hardness measurements were carried out using a Shore D hardness tester according to ASTM D2240 [37]. Shore D hardness measurements were performed using an LX-D-2 dual-needle durometer (HUATEC Group Corporation, Beijing, China) to assess the hardness characteristics of the prepared epoxy-based biocomposites. Measurements were performed at multiple locations on each specimen to minimize local heterogeneity effects. The reported hardness values represent the arithmetic mean of repeated measurements [36].

### 2.6. Tensile Test

Tensile properties of the fabricated composites were determined according to ASTM D638 [38]. The tensile properties of the produced EBCs were evaluated using a universal testing machine (UTEST, Ankara, Türkiye) with a maximum load capacity of 1 kN. Dog-bone-shaped specimens were prepared using silicone molds. Tensile tests were performed using a universal testing machine equipped with an appropriate load cell. Tensile strength, and elongation at break were obtained directly from stress–strain curves generated during testing. At least five specimens were tested for each formulation, and average values were reported [39].

### 2.7. Dielectric Constant

The dielectric properties of the fabricated epoxy-based biocomposites were measured using a Fytronix precision LCR meter (Fytronix, Elazığ, Türkiye) over a frequency range of 1 kHz to 1 MHz in accordance with ASTM D150 [40]. Circular specimens with uniform thickness were prepared for dielectric measurements. Dielectric constant and dielectric loss values were determined as functions of frequency. The influence of graphene nanoplatelet concentration on dielectric behavior was systematically evaluated [41].

### 2.8. Thermal Conductivity Analysis

Thermal conductivity measurements were carried out using a steady-state heat flow thermal conductivity analyzer (TLS-100, Thermtest Inc., Fredericton, NB, Canada). The analysis was performed to investigate the effect of GNP incorporation on the thermal transport behavior of the improved EBCs. Thermal conductivity coefficients of the prepared composites were measured using a thermal conductivity analyzer operating under steady-state conditions. Prior to testing, specimens were conditioned at laboratory temperature and humidity. Measurements were performed on multiple specimens to ensure reproducibility. Average thermal conductivity values were calculated and reported [42].

### 2.9. Thermogravimetric Analysis (TGA)

Thermal degradation behavior was investigated using a Hitachi STA 200 brand TGA (Hitachi High-Tech Corporation, Tokyo, Japan). Approximately 10 ± 1 mg of each specimen was placed in an alumina crucible and heated from 30 to 600 °C at a constant heating rate of 10 °C min^−1^ under a nitrogen atmosphere flowing at 50 mL min^−1^. The mass loss, degradation profile, and residual char content were continuously recorded throughout the experiment [43].

### 2.10. FTIR Analysis

Chemical interactions between the epoxy matrix, CSP, and GNP were investigated. FTIR spectroscopy was carried out using a Shimadzu IRSpirit QATR-S spectrometer (Shimadzu, Kyoto, Japan) to obtain high-resolution infrared spectra and to identify the characteristic functional groups and chemical interactions within the developed EBCs. Spectra were collected within the range of 4000–400 cm^−1^. The characteristic functional groups associated with epoxy, lignocellulosic components, and graphene structures were identified and compared among different formulations [44].

### 2.11. SEM and EDX Analysis

The surface morphology and microstructural characteristics of the produced EBCs were examined using a Zeiss EVO MA 10 SEM (ZEISS, Oberkochen, Germany), which enabled the acquisition of high-resolution micrographs for detailed morphological analysis. The fracture surfaces of tensile-tested specimens were examined using scanning electron microscopy to evaluate filler dispersion, interfacial adhesion, void formation, crack propagation, and fracture mechanisms. Prior to imaging, all specimens were sputter-coated with a thin conductive gold layer to minimize charging effects. Elemental composition and elemental mapping were analyzed using an integrated energy-dispersive X-ray spectroscopy (EDX) detector (EDAX, Mahwah, NJ, USA) operating under identical accelerating voltage conditions. Carbon, oxygen, and other detectable elements were quantified to verify the successful incorporation and distribution of the reinforcing phases [39].

## 3. Results and Discussion

### 3.1. Bulk Density Results

**Figure 2:**
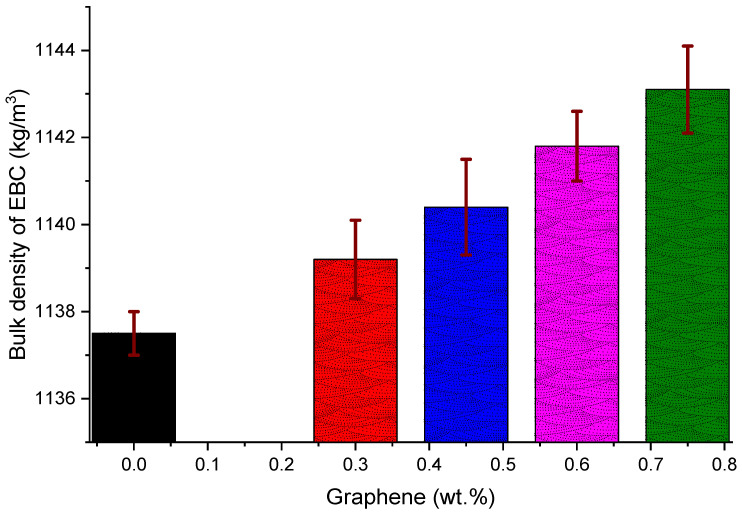
Effect of GNP content on bulk density of EBCs.

Figure 2 illustrates the variation in the bulk density of the epoxy-based coconut shell biocomposites as a function of GNP content. The bulk density of the graphene-free composite (EBC 1) was measured as 1137.5 ± 0.5 kg/m^3^. Upon the incorporation of graphene nanoplatelets, a gradual and nearly linear increase in bulk density was observed. The density values increased to 1139.2 ± 0.9 kg/m^3^, 1140.4 ± 1.1 kg/m^3^, 1141.8 ± 0.8 kg/m^3^, and 1143.1 ± 1.0 kg/m^3^ for EBC 2, EBC 3, EBC 4, and EBC 5.

The increase in density can be primarily attributed to the inherently high density of graphene nanoplatelets compared with both the epoxy matrix and the lignocellulosic coconut shell filler. Graphene possesses a highly ordered carbon structure and a considerably higher intrinsic density than the surrounding polymeric phase. Therefore, even at relatively low loading levels (0.30–0.75 wt.%), the addition of graphene contributes to a measurable increase in the overall density of the composite system. Furthermore, the nanoscale dimensions and high specific surface area of graphene nanoplatelets promote efficient packing within the polymer network, reducing microvoid formation and improving structural compactness [45].

Compared with EBC 1, the density of EBC 5 increased by approximately 0.49%, indicating that graphene loading had a positive but moderate effect on the bulk density of the composites. This relatively small increase is expected because the graphene content remained below 1 wt.% throughout the study. Similar trends have been reported for graphene-reinforced thermosetting polymers, where low graphene concentrations improve packing efficiency and increase density without causing significant changes in composite weight.

Another important observation is the low standard deviation values obtained for all samples, ranging from ±0.5 to ±1.1 kg/m^3^. These small deviations indicate good repeatability of the fabrication process and suggest a homogeneous distribution of both coconut shell particles and graphene nanoplatelets within the epoxy matrix. The absence of large fluctuations also implies that no significant agglomeration of graphene occurred during processing, which is crucial for achieving consistent composite properties.

The gradual increase in density further suggests that graphene nanoplatelets were successfully incorporated into the epoxy network and occupied the interstitial regions between the epoxy matrix and coconut shell particles. Such a densification effect is beneficial because improved packing generally enhances interfacial adhesion and load transfer efficiency between the matrix and reinforcing phases. The observed density increase may contribute positively to the mechanical and thermal performance of the developed biocomposites [46].

The results demonstrate that the incorporation of GNPs led to a systematic increase in bulk density, with the highest density recorded for EBC 5 (1143.1 kg/m^3^). Although the magnitude of the increase was relatively limited due to the low graphene loading, the trend confirms the effective integration of graphene within the coconut shell-filled epoxy matrix and indicates the formation of a more compact composite structure. Such behavior is advantageous for the development of high-performance bio-based epoxy composites intended for structural, thermal, and multifunctional engineering applications.

### 3.2. Shore D Hardness Results

**Figure 3:**
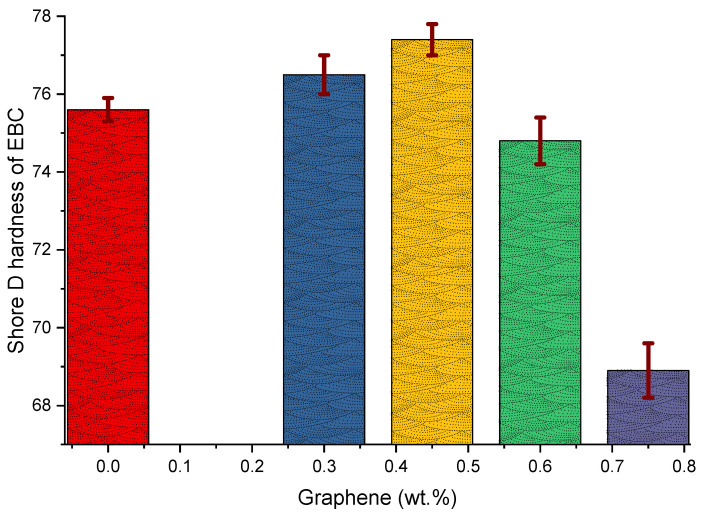
Effect of GNP content on Shore D hardness of EBCs.

Figure 3 illustrates the variation in Shore D hardness of EBCs as a function of GNP loading. The Shore D hardness increased from 75.6 ± 0.3 for EBC 1 (0 wt.% GNP) to 76.5 ± 0.5 and 77.4 ± 0.4 for EBC 2 (0.30 wt.% GNP) and EBC 3 (0.45 wt.% GNP). The highest hardness value was obtained at 0.45 wt.% GNP, corresponding to an increase of approximately 2.4% compared with the GNP-free composite. This improvement can be attributed to the exceptional intrinsic stiffness and mechanical strength of graphene nanopowder, which restricts polymer chain mobility and enhances the rigidity of the epoxy matrix. Furthermore, the homogeneous dispersion of low concentrations of GNP likely promotes efficient stress transfer between the CSP and the surrounding epoxy network, resulting in improved surface resistance against indentation [47].

However, further increasing the GNP content beyond 0.45 wt.% led to a noticeable decline in hardness. The Shore D hardness decreased to 74.8 ± 0.6 at 0.60 wt.% GNP and dramatically dropped to 68.9 ± 0.7 at 0.75 wt.% GNP. This reduction suggests that excessive GNP loading adversely affected the microstructure of the composites. At higher concentrations, graphene nanoparticles tend to agglomerate due to strong van der Waals interactions and their large specific surface area. These agglomerates can act as stress concentration sites and hinder the formation of a uniform cross-linked epoxy network, thereby reducing the effectiveness of reinforcement. In addition, nanoparticle clustering may introduce microvoids and weak interfacial regions within the composite structure, resulting in decreased hardness.

The obtained results indicate that the reinforcing efficiency of GNP is highly dependent on its concentration. While low GNP loadings effectively enhance the hardness of the coconut shell-reinforced epoxy composites, excessive nanoparticle incorporation leads to particle agglomeration and deterioration of mechanical performance. Therefore, 0.45 wt.% GNP can be considered the optimum loading level for maximizing the Shore D hardness of the EBCs investigated in this study. The observed trend demonstrates the critical role of nanoparticle dispersion quality in determining the final mechanical properties of graphene-modified epoxy biocomposites.

### 3.3. Tensile Test Results

**Figure 4:**
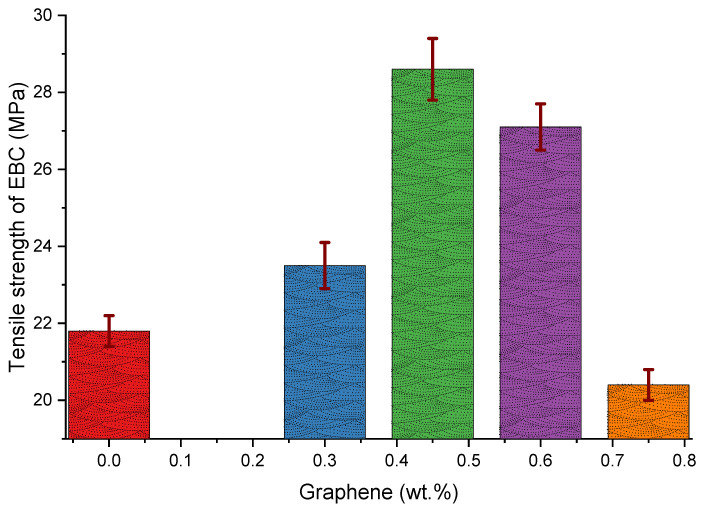
Effect of GNP content on the tensile strength of EBCs.

Figure 4 shows the variation in tensile strength of EBCs reinforced with coconut shell particles as a function of GNP content. The tensile strength increased from 21.8 ± 0.4 MPa for the GNP-free composite (EBC 1) to 23.5 ± 0.6 MPa and 28.6 ± 0.8 MPa for EBC 2 and EBC 3 containing 0.30 and 0.45 wt.% GNP. The maximum tensile strength was achieved at 0.45 wt.% GNP, corresponding to an enhancement of approximately 31.2% compared with EBC 1. This result indicates that a moderate amount of GNP can significantly improve the load-bearing capacity of the EBC.

The improvement observed at low GNP concentrations can be attributed to the excellent mechanical properties of graphene and its ability to reinforce the epoxy matrix. When uniformly dispersed, GNP provides efficient stress-transfer pathways between the coconut shell particles and the surrounding epoxy network. Furthermore, the large surface area of graphene enhances interfacial adhesion, allowing external loads to be distributed more effectively throughout the composite structure. Crack initiation and propagation are inhibited, resulting in higher tensile strength values.

The highest tensile strength obtained for EBC 3 suggests that 0.45 wt.% GNP represents the optimum nanoparticle loading for the investigated system. At this concentration, GNP is likely well dispersed within the matrix and contributes effectively to reinforcement without significantly disturbing the epoxy crosslinking network. The strong filler–matrix interaction and improved stress transfer efficiency are responsible for the superior mechanical performance observed at this composition [48].

However, increasing the GNP content beyond the optimum level resulted in a reduction in tensile strength. The tensile strength decreased to 27.1 ± 0.6 MPa for EBC 4 containing 0.60 wt.% GNP and dropped markedly to 20.4 ± 0.4 MPa for EBC 5 containing 0.75 wt.% GNP. The reduction in strength at higher GNP concentrations can be attributed to nanoparticle agglomeration. Due to strong van der Waals interactions between graphene sheets, excessive GNP loading promotes the formation of agglomerates that act as stress concentration sites within the composite. These localized defects weaken the interfacial bonding and facilitate premature crack formation under tensile loading.

The substantial decrease observed for EBC 5 indicates that the detrimental effects of agglomeration outweighed the reinforcing contribution of graphene at high filler concentrations. In addition, graphene clusters may hinder homogeneous stress distribution and reduce the effectiveness of the epoxy matrix in transferring loads. Such behavior is commonly reported in graphene-reinforced polymer nanocomposites when the nanoparticle concentration exceeds the optimum dispersion threshold.

The results demonstrate that the tensile properties of the developed EBCs are strongly dependent on GNP loading. The incorporation of a small amount of GNP significantly enhanced tensile strength, whereas excessive loading led to agglomeration-induced deterioration of mechanical performance. Among the investigated formulations, EBC 3 containing 0.45 wt.% GNP exhibited the best tensile performance, indicating the existence of an optimum graphene concentration for achieving maximum reinforcement in coconut shell-reinforced epoxy biocomposites.

**Figure 5:**
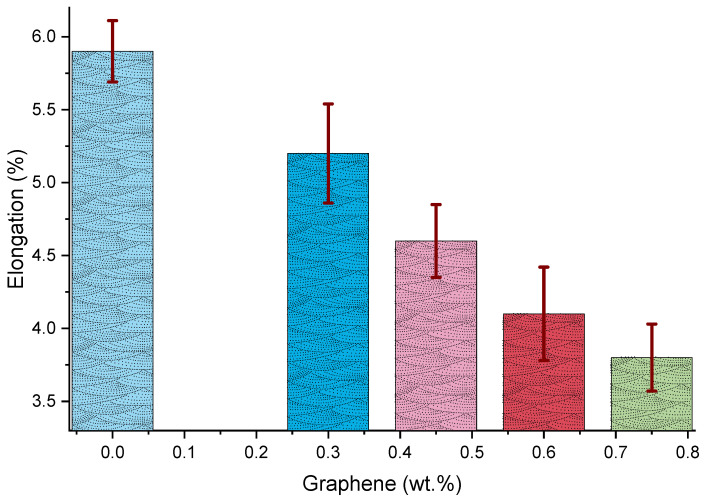
Effect of GNP content on the elongation at break of EBC.

Figure 5 presents the effect of GNP loading on the elongation at break of EBCs reinforced with coconut shell particles. The elongation at break exhibited a continuous decline with increasing GNP content, decreasing from 5.9 ± 0.21% for the GNP-free composite (EBC 1) to 5.2 ± 0.34%, 4.6 ± 0.25%, 4.1 ± 0.32%, and 3.8 ± 0.23% for EBC 2, EBC 3, EBC 4, and EBC 5. Compared with EBC 1, the elongation at break of EBC 5 decreased by approximately 35.6%, indicating a progressive reduction in ductility as the GNP concentration increased.

The reduction in elongation can be primarily attributed to the rigid and highly stiff nature of GNP. When incorporated into the epoxy matrix, GNP restricts the mobility of polymer chains and limits molecular rearrangement during tensile deformation. Consequently, the ability of the composite to undergo plastic deformation before fracture decreases. Although graphene contributes positively to mechanical reinforcement, it simultaneously reduces the flexibility of the polymer network, leading to lower elongation values.

At low GNP concentrations (0.30–0.45 wt.%), the decrease in elongation was relatively moderate. This behavior suggests that GNP was reasonably dispersed within the matrix and effectively reinforced the composite without severely compromising its deformation capability. The corresponding increase in tensile strength observed in the figure further confirms that the reinforcing effect of graphene dominated at these concentrations. In particular, EBC 3 containing 0.45 wt.% GNP exhibited the highest tensile strength while still maintaining an elongation value of 4.6%, indicating a favorable balance between strength and ductility.

As the GNP loading increased beyond 0.45 wt.%, a more pronounced reduction in elongation was observed. The elongation decreased to 4.1% for EBC 4 and further to 3.8% for EBC 5. This behavior may be associated with the formation of graphene agglomerates at higher nanoparticle concentrations. Such agglomerates can act as stress concentration sites, facilitating crack initiation and accelerating crack propagation during tensile loading. As a result, the composites fail at lower strain levels and exhibit increasingly brittle behavior.

The gradual decrease in elongation is also consistent with the observed increases in Shore D hardness, bulk density, dielectric constant, and thermal conductivity. These results collectively indicate that GNP enhances the structural rigidity and multifunctional performance of the epoxy-based biocomposites while reducing their ability to deform elastically and plastically. The increase in stiffness and intermolecular constraints within the epoxy network inevitably limits chain mobility, resulting in reduced elongation at break [19,48].

The results demonstrate that GNP significantly influences the deformation behavior of the developed EBCs. While increasing GNP loading improves several functional and mechanical properties, it simultaneously decreases ductility. Among the investigated formulations, EBC 3 (0.45 wt.% GNP) provided the most balanced performance, combining the highest tensile strength with a relatively acceptable elongation at break. Therefore, this composition may be considered the optimum formulation for applications requiring both mechanical reinforcement and adequate deformation capability.

**Figure 6:**
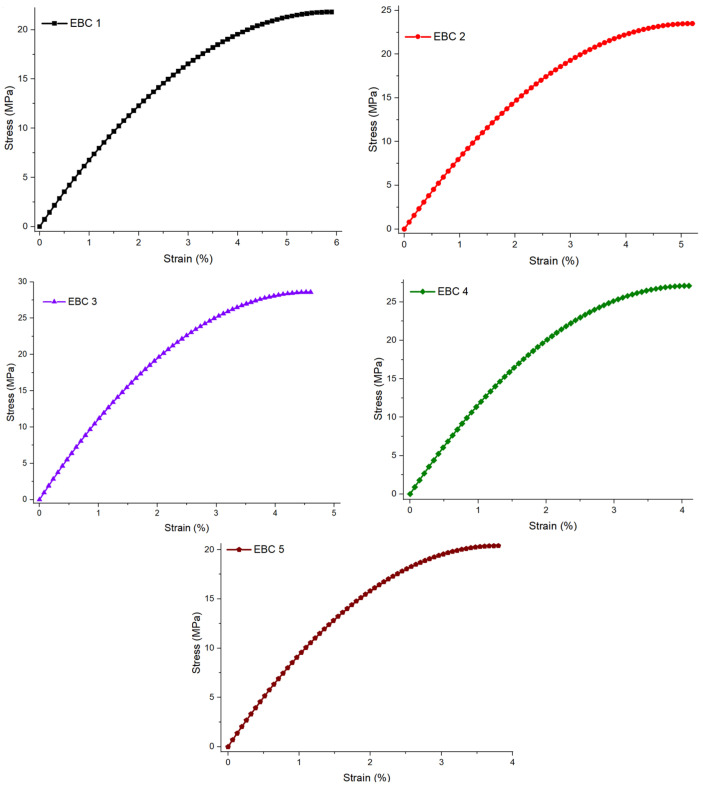
Effect of GNP content on the elongation at break of EBCs.

Figure 6 presents the representative stress–strain curves of the epoxy-based biocomposites reinforced with CSP and different GNP loadings. The tensile strength increased from 21.8 ± 0.4 MPa for EBC 1 to 23.5 ± 0.6 MPa and reached a maximum value of 28.6 ± 0.8 MPa for EBC 3 containing 0.45 wt.% GNP, corresponding to an improvement of approximately 31.2% compared with the control sample. This enhancement is attributed to the uniform dispersion of GNP within the epoxy matrix, which improves interfacial adhesion between the matrix and CSPs, promotes efficient stress transfer, and delays crack initiation during tensile loading. However, a further increase in GNP content resulted in a gradual deterioration of tensile performance, with the tensile strength decreasing to 27.1 ± 0.6 MPa for EBC 4 and 20.4 ± 0.4 MPa for EBC 5. Simultaneously, the elongation at break continuously decreased from 5.9% for EBC 1 to 5.2%, 4.6%, 4.1%, and 3.8% for EBC 2–EBC 5, demonstrating a progressive reduction in ductility as the graphene content increased. The lower elongation values indicate that GNP effectively restricted the mobility of epoxy molecular chains and increased the stiffness of the composite network. At the highest GNP loading (0.75 wt.%), the reduction in both tensile strength and elongation suggests that the reinforcing effect of graphene was outweighed by adverse microstructural phenomena, such as nanoparticle agglomeration, localized stress concentration, and reduced dispersion quality, which weakened the filler–matrix interface and facilitated premature crack propagation. The stress–strain curves clearly indicate that 0.45 wt.% GNP represents the optimum reinforcement level for achieving the best balance between strength and ductility, confirming that the mechanical performance of the developed epoxy/CSP hybrid biocomposites is strongly governed by graphene dispersion quality and interfacial interactions within the composite structure.

### 3.4. Dielectric Constant Results

**Figure 7:**
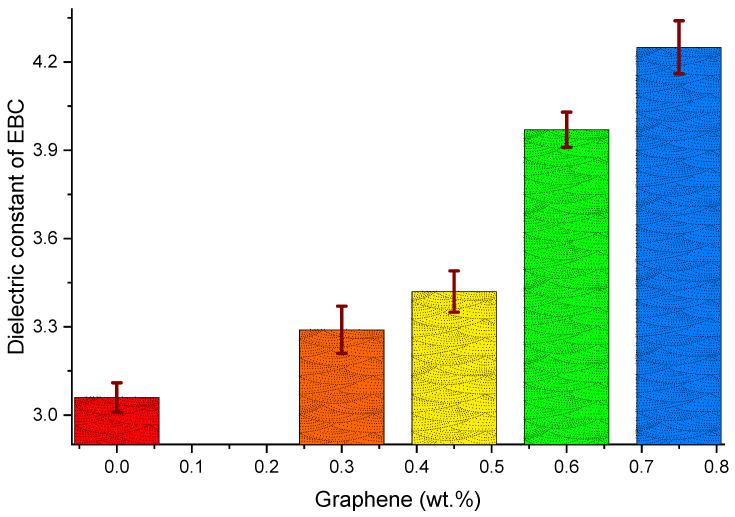
Effect of GNP content on the dielectric constant of EBCs.

Figure 7 presents the variation in the dielectric constant of EBC composites as a function of GNP loading. The dielectric constant increased progressively from 3.06 ± 0.05 for the GNP-free composite (EBC 1) to 3.29 ± 0.08, 3.42 ± 0.07, 3.97 ± 0.06, and 4.25 ± 0.09 for EBC 2, EBC 3, EBC 4, and EBC 5. Compared with EBC 1, the dielectric constant of EBC 5 increased by approximately 38.9%, demonstrating the significant influence of GNP incorporation on the electrical polarization behavior of the composites.

The gradual increase in dielectric constant with increasing GNP content can be attributed to the outstanding electrical characteristics and extremely high specific surface area of GNP. The introduction of conductive graphene nanosheets into the insulating epoxy matrix promotes the formation of numerous micro-capacitive regions and enhances charge accumulation at the interfaces between the epoxy matrix, CSPs, and GNPs [49].

At low GNP loadings (0.30–0.45 wt.%), a moderate increase in dielectric constant was observed, indicating that the graphene particles were well dispersed within the polymer matrix and contributed primarily through interfacial polarization mechanisms. However, a more pronounced increase occurred between 0.45 wt.% and 0.60 wt.% GNP, where the dielectric constant increased from 3.42 to 3.97. This behavior suggests enhanced connectivity between graphene particles and a substantial increase in the number of polarization centers within the composite structure.

The highest dielectric constant was obtained for EBC 5 (0.75 wt.% GNP). At this concentration, the GNP provided the largest interfacial area and the highest density of localized charge carriers, resulting in enhanced dielectric polarization. Although the GNP content remained below the percolation threshold, the proximity between conductive graphene particles facilitated charge storage and increased the effective permittivity of the composite.

The obtained results clearly demonstrate that GNP is an effective dielectric modifier for coconut shell-reinforced epoxy composites. The continuous increase in dielectric constant with increasing GNP loading indicates strong interfacial interactions among the epoxy matrix, lignocellulosic CSPs, and GNPs. Therefore, the incorporation of GNP offers a promising strategy for developing sustainable EBCs with enhanced dielectric performance for potential applications in electrical insulation systems, dielectric layers, electronic packaging, sensors, and multifunctional structural materials.

### 3.5. Thermal Conductivity Results

**Figure 8:**
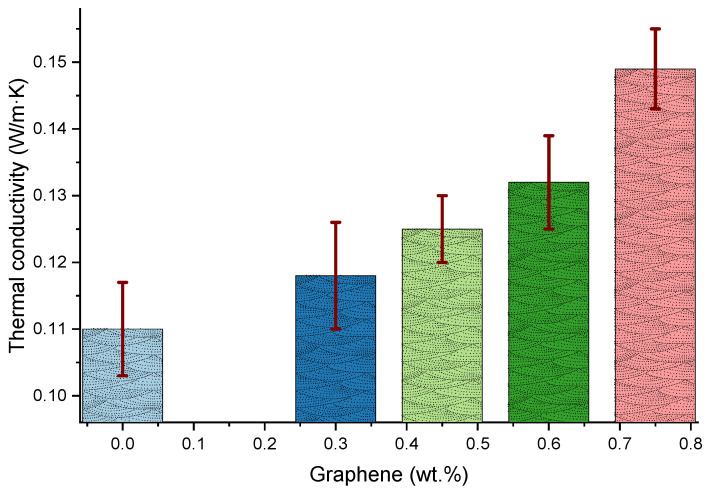
Effect of GNP content on the thermal conductivity of EBCs.

Figure 8 illustrates the influence of GNP loading on the thermal conductivity of EBCs reinforced with CSPs. The thermal conductivity increased steadily from 0.110 ± 0.007 W/m·K for EBC 1 to 0.118 ± 0.008 W/m·K, 0.125 ± 0.005 W/m·K, 0.132 ± 0.007 W/m·K, and 0.149 ± 0.006 W/m·K for EBC 2, EBC 3, EBC 4, and EBC 5. Compared with EBC 1, the thermal conductivity of EBC 5 increased by approximately 35.5%, demonstrating the remarkable effectiveness of GNP as a thermally conductive nanofiller within the biocomposite structure.

The relatively low thermal conductivity observed for EBC 1 can be attributed to the presence of CSP, which consist primarily of lignocellulosic components such as cellulose, hemicellulose, and lignin. These constituents inherently possess low thermal conductivity and tend to hinder heat transfer through the composite matrix. Consequently, the GNP-free biocomposite exhibited a thermal conductivity slightly lower than or comparable to that of neat epoxy systems reported in the literature [50].

The incorporation of GNP significantly enhanced thermal transport within the epoxy matrix. Owing to its exceptionally high intrinsic thermal conductivity, graphene acts as an efficient heat-conducting medium and facilitates phonon transport through the composite. At low GNP loadings (0.30–0.45 wt.%), a gradual increase in thermal conductivity was observed, indicating that the graphene nanosheets were sufficiently dispersed and capable of creating localized heat-conduction pathways throughout the matrix. The increase from 0.110 W/m·K to 0.125 W/m·K confirms the positive contribution of well-dispersed graphene to thermal energy transfer.

A further increase in GNP content resulted in a more pronounced enhancement of thermal conductivity. The values obtained for EBC 4 and EBC 5 suggest the development of increasingly interconnected thermally conductive networks within the composite structure. As the distance between adjacent graphene particles decreases, thermal resistance at the filler–matrix interface is reduced, allowing heat to propagate more efficiently through the epoxy matrix. The formation of these conductive pathways is responsible for the substantial increase in thermal conductivity observed at higher GNP concentrations.

The highest thermal conductivity was achieved for EBC 5 containing 0.75 wt.% GNP, reaching 0.149 ± 0.006 W/m·K. This value indicates that even a relatively small amount of GNP can significantly improve the heat-transfer capability of CSP-reinforced epoxy biocomposites. The enhancement is attributed to the synergistic effect of graphene’s high intrinsic thermal conductivity and the improved connectivity between thermally conductive regions within the polymer matrix.

The results demonstrate that graphene nanopowder is a highly effective nanoreinforcement for improving the thermal performance of epoxy-based biocomposites. The continuous increase in thermal conductivity with increasing GNP loading indicates that GNP successfully compensates for the inherently low thermal conductivity of lignocellulosic CSP fillers. Therefore, the developed EBCs exhibit considerable potential for applications requiring lightweight and sustainable materials with enhanced thermal management capabilities, including electronic packaging, thermal insulation components with controlled heat dissipation, multifunctional structural composites, and advanced engineering materials.

### 3.6. TGA Results

**Figure 9:**
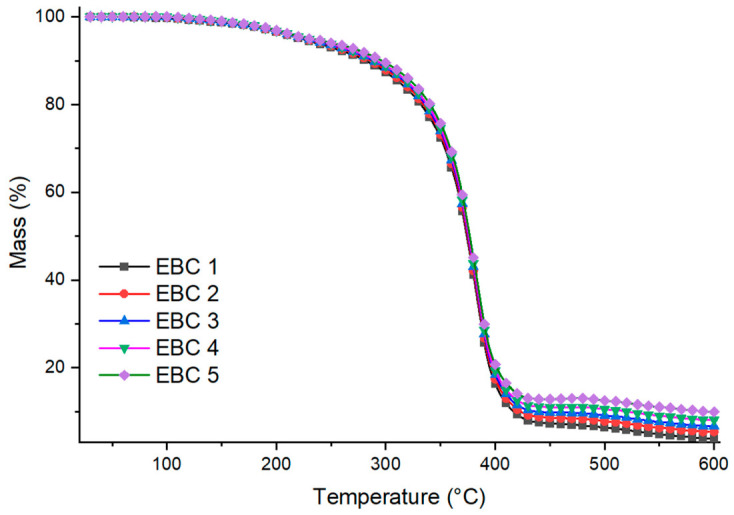
TGA result of EBCs.

Figure 9 presents the TGA curves of the EBCs reinforced with a constant amount of coconut shell filler and different GNP loadings. The thermal decomposition behavior clearly demonstrates that the incorporation of GNP improves the thermal stability of the composites, whereas the coconut shell filler alone causes a slight reduction in thermal resistance compared with neat epoxy systems reported in the literature [51].

The TGA curves reveal that all EBC formulations exhibit a similar degradation profile characterized by three distinct stages. The first stage, occurring below approximately 200 °C, corresponds to the evaporation of physically adsorbed moisture, residual volatiles, and low-molecular-weight compounds trapped within the composite structure. During this stage, all samples exhibited negligible mass loss, retaining more than 96% of their original mass at 200 °C. The similarity of the curves in this region indicates that the addition of GNP does not significantly affect the moisture content of the composites.

The second degradation stage, extending from approximately 250 °C to 420 °C, represents the principal thermal decomposition region of the epoxy network. In this temperature range, cleavage of ether linkages, decomposition of hydroxyl-containing structures, scission of C–N bonds, and degradation of the cross-linked epoxy matrix occur simultaneously. As shown in Figure 9, the degradation rate increased sharply above 320 °C, leading to substantial mass losses for all specimens. However, the extent of degradation strongly depended on the GNP content. At 380 °C, the residual mass values were 41.15, 42.10, 43.00, 43.80, and 45.10% for EBC 1, EBC 2, EBC 3, EBC 4, and EBC 5. Similarly, at 400 °C the remaining mass increased from 16.35% for EBC 1 to 20.70% for EBC 5, indicating a progressive enhancement in thermal stability with increasing graphene content.

The improved thermal resistance can be attributed to the unique barrier effect of GNP. The high aspect ratio and excellent thermal resistance of graphene create tortuous pathways that restrict the diffusion of volatile degradation products and oxygen throughout the polymer matrix. Consequently, heat transfer into the bulk material becomes slower, delaying thermal decomposition and reducing the degradation rate. Furthermore, strong interfacial interactions between the epoxy matrix and graphene nanosheets contribute to a more thermally stable network structure.

A comparison of the degradation behavior around the maximum decomposition region further confirms the beneficial effect of GNP. At 350 °C, EBC 1 retained 72.40% of its original mass, whereas EBC 5 retained 75.70%. The difference became more pronounced at higher temperatures, reaching approximately 4–5 percentage points in the 380–430 °C range. This observation indicates that graphene is particularly effective during the main degradation stage, where the formation of a thermally resistant carbonaceous layer acts as a protective shield against further decomposition.

The final degradation stage occurred above approximately 450 °C, where decomposition slowed considerably and a relatively stable carbonaceous residue remained. The residual mass at 600 °C increased systematically with increasing GNP content. EBC 1 exhibited the lowest char residue (3.86%), while EBC 2, EBC 3, EBC 4, and EBC 5 showed residual masses of 5.40, 6.70, 8.10, and 9.90%. The residual mass of EBC 5 was therefore approximately 2.6 times higher than that of EBC 1. This significant increase in char yield confirms the role of graphene in promoting carbonization and suppressing the release of volatile degradation products during thermal decomposition.

The gradual increase in char formation suggests that GNP acts not only as a reinforcing nanofiller but also as an effective thermal stabilizer. During decomposition, graphene sheets facilitate the formation of a compact and continuous protective carbon layer that limits heat penetration and volatile transport. Such behavior is highly desirable for applications requiring enhanced thermal resistance, dimensional stability, and flame retardancy.

The TGA results demonstrate that increasing the GNP content significantly improves the thermal stability of the EBCs. Although the coconut shell filler introduces a slight reduction in thermal resistance compared with pure epoxy systems, the incorporation of GNP effectively compensates for this effect and substantially enhances thermal performance. Among all formulations, EBC 5 containing 0.75 wt.% GNP exhibited the highest thermal stability, highest residual mass, and greatest resistance to thermal degradation, indicating that this composition provides the most thermally robust structure among the investigated composites.

### 3.7. FTIR Spectra Results

**Figure 10:**
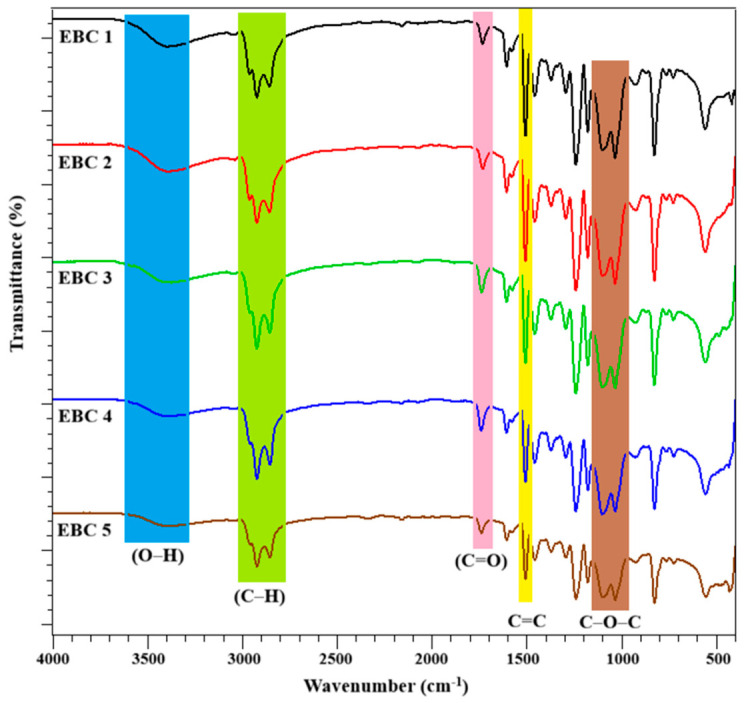
FTIR spectra of EBCs reinforced with CSP and GNP.

Figure 10 shows the FTIR spectra of the EBCs containing a constant amount of coconut shell powder and increasing GNP content. The spectra exhibit the characteristic absorption bands of the cured epoxy network together with the lignocellulosic functional groups originating from coconut shell particles. The overall similarity of the spectra indicates that the incorporation of GNP did not introduce new chemical bonds but mainly affected the intensity and sharpness of several existing absorption bands. This observation suggests that the interaction between GNP, coconut shell particles, and the epoxy matrix is predominantly physical rather than chemical [52].

A broad absorption band observed in the region of approximately 3520–3310 cm^−1^ is attributed to the stretching vibration of hydroxyl (O–H) groups. These hydroxyl groups originate primarily from cellulose, hemicellulose, lignin, and residual hydroxyl functionalities present within the cured epoxy network. The presence of this band confirms the lignocellulosic nature of the CSPs. A slight reduction in band intensity with increasing GNP loading suggests the formation of intermolecular hydrogen-bonding interactions between graphene surfaces and hydroxyl-containing components of the composite structure. Such interactions may contribute to improved interfacial adhesion between the reinforcing phases and the epoxy matrix [53].

The absorption peaks located near 2920 cm^−1^ and 2850 cm^−1^ correspond to asymmetric and symmetric stretching vibrations of aliphatic C–H bonds associated with methylene and methyl groups present in both the epoxy resin and coconut shell constituents. These peaks remain visible in all formulations, indicating that the fundamental hydrocarbon structure of the polymer matrix was preserved after graphene incorporation. The relatively unchanged position of these bands further confirms that no significant chemical degradation or structural alteration occurred during composite fabrication.

A weak absorption band observed around 1730–1700 cm^−1^ can be assigned to carbonyl (C=O) stretching vibrations associated with ester, aldehyde, ketone, or carboxylic functionalities originating from lignin and hemicellulose components of coconut shell particles. The relatively low intensity of this peak indicates that oxygen-containing carbonyl groups constitute only a minor fraction of the composite structure. Nevertheless, their presence confirms the successful incorporation of the lignocellulosic biofiller into the epoxy matrix.

The spectral region between 1600 and 1500 cm^−1^ contains several characteristic absorption peaks attributed to aromatic skeletal vibrations. The band near 1600 cm^−1^ is associated with aromatic C=C stretching vibrations originating from both the bisphenol-A-based epoxy resin and the aromatic structures present in lignin. In addition, peaks around 1510–1450 cm^−1^ correspond to aromatic ring vibrations and methylene bending modes. A slight increase in the intensity of these peaks with increasing GNP content may be attributed to the graphitic aromatic structure of GNP, which contributes additional sp^2^-hybridized carbon domains to the composite system.

One of the most prominent spectral regions appears between 1250 and 1000 cm^−1^, where several strong absorption bands are observed. These bands are mainly assigned to C–O–C stretching, ether linkages, epoxy ring-derived structures, and C–O stretching vibrations of cellulose and hemicellulose. The strong peaks near 1230 cm^−1^, 1160 cm^−1^, and 1030 cm^−1^ confirm the existence of ether bonds within the crosslinked epoxy network as well as oxygen-containing functional groups derived from coconut shell powder. The persistence of these peaks across all formulations indicates that the epoxy curing process proceeded successfully regardless of graphene concentration.

The absorption bands detected in the region of approximately 900–820 cm^−1^ are associated with aromatic C–H out-of-plane bending vibrations and residual epoxy-related structural units. The relatively weak intensity of these bands indicates a highly cured epoxy network, suggesting that most reactive epoxy groups participated in the crosslinking reaction. Furthermore, no significant peak shifts were observed in this region after graphene addition, confirming that GNP did not chemically interfere with the curing mechanism of the epoxy resin.

At lower wavenumbers, particularly below 800 cm^−1^, several minor absorption bands are visible and can be attributed to aromatic ring deformation, skeletal vibrations of lignin structures, and graphitic carbon-related vibrations. The slight enhancement of these bands in EBC 4 and EBC 5 may be associated with the increasing concentration of GNP and its contribution to the overall carbonaceous structure of the composites.

A comparison of all spectra demonstrates that the characteristic absorption bands remain located at nearly identical wavenumbers for EBC 1–EBC 5. No new peaks appear and no major peak shifts are detected with increasing graphene content. Such behavior indicates that GNP acts primarily as a physical nano reinforcement dispersed within the epoxy–coconut shell matrix rather than participating directly in chemical reactions. Nevertheless, subtle changes in peak intensity suggest enhanced interfacial interactions and improved compatibility between the composite constituents.

The FTIR results confirm the successful fabrication of coconut shell/graphene-reinforced EBCs. The spectra verify the coexistence of characteristic functional groups originating from the cured epoxy matrix, lignocellulosic CSP, and GNP. The absence of significant spectral changes indicates that graphene incorporation does not alter the fundamental chemical structure of the epoxy network, while the observed variations in peak intensity suggest improved interfacial interactions that may contribute to the enhanced mechanical, thermal, and dielectric properties observed in the developed EBCs [54].

### 3.8. SEM and EDX Analysis Results

**Figure 11:**
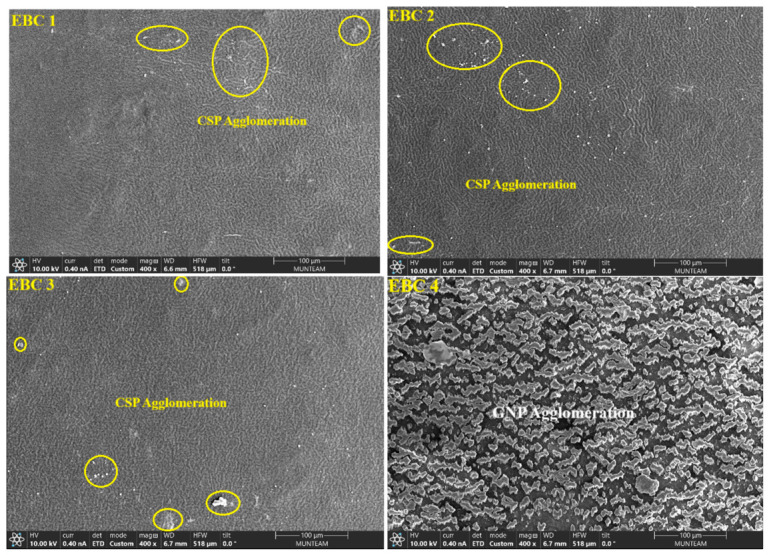
SEM images of EBCs reinforced with CSP and GNP.

Figure 11 shows the SEM images of the fractured surfaces of EBCs containing a constant amount of CSP and different GNP concentrations. The micrographs provide valuable information regarding filler dispersion, surface morphology, interfacial adhesion, structural homogeneity, and the influence of graphene addition on the microstructural evolution of the composites [55,56,57,58,59].

The SEM image of EBC 1 exhibits a relatively smooth and homogeneous fracture surface with limited surface roughness. The morphology is characteristic of a brittle thermosetting epoxy matrix reinforced only with CSP. Several small micro-irregularities and localized rough regions can be observed, which are associated with the presence of lignocellulosic CSP embedded within the cured epoxy network. However, no significant agglomerated structures are visible, indicating that the coconut shell powder was reasonably dispersed throughout the matrix. The relatively smooth fracture surface suggests limited energy absorption during crack propagation, which is consistent with the lower tensile strength and lower hardness values observed for EBC 1. The absence of graphene also results in fewer obstacles to crack growth, allowing cracks to propagate more easily through the epoxy matrix [56,57,58,59].

The SEM micrograph of EBC 2 reveals a noticeably rougher surface compared with EBC 1. The fracture surface exhibits more pronounced micro-ridges, wrinkles, and localized deformation features. Such morphological changes indicate improved interaction between the epoxy matrix and reinforcing phases. The introduction of a small amount of GNP appears to enhance interfacial bonding and restrict crack propagation. Fine bright regions dispersed throughout the microstructure may correspond to graphene-rich domains uniformly distributed within the epoxy matrix. No significant graphene agglomeration is observed at this concentration, suggesting effective nanoparticle dispersion. The improved surface roughness indicates increased fracture energy absorption and stronger resistance to crack initiation, which correlates well with the increase in tensile strength, Shore D hardness, and density reported for EBC 2.

The fracture morphology of EBC 3 demonstrates the most uniform and compact microstructure among all investigated samples. The surface appears highly homogeneous, with well-distributed microstructural features and minimal evidence of void formation or particle pull-out. The GNP seems to be finely dispersed throughout the epoxy matrix, producing a dense interconnected structure. The absence of large defects indicates excellent compatibility between GNP, CSP, and the epoxy resin. Furthermore, the increased roughness and tortuous fracture path suggest that propagating cracks encountered numerous obstacles during failure. This phenomenon increases crack deflection, crack pinning, and energy dissipation mechanisms. EBC 3 exhibits the highest tensile strength and nearly the highest Shore D hardness among all formulations. The superior mechanical performance observed experimentally is therefore strongly supported by the microstructural characteristics revealed in the SEM image.

A significantly different morphology is observed for EBC 4. The fracture surface becomes considerably rougher and more heterogeneous, displaying numerous irregular interconnected structures distributed throughout the matrix. While the increased roughness initially suggests enhanced reinforcement, closer examination indicates the emergence of localized graphene-rich clusters and microstructural heterogeneity. These regions are likely associated with the beginning of graphene agglomeration resulting from strong van der Waals interactions between adjacent graphene sheets. The formation of such agglomerates may partially disrupt the continuity of the epoxy network and generate localized stress concentration zones. Although graphene still contributes positively to thermal conductivity, dielectric properties, and thermal stability, the appearance of these heterogeneous regions may explain the slight reduction in tensile strength and Shore D hardness observed for EBC 4 compared with EBC 3.

A comparative evaluation of the SEM micrographs clearly demonstrates the influence of graphene concentration on composite morphology. The transition from the relatively smooth fracture surface of EBC 1 to the highly compact structure of EBC 3 indicates progressive improvement in filler dispersion and interfacial adhesion. At low and moderate graphene contents, the nanopowder effectively occupies microvoids within the epoxy matrix, promotes stress transfer between phases, and increases structural compactness. This observation is consistent with the measured increases in bulk density, tensile strength, thermal conductivity, and dielectric constant. However, when the graphene concentration exceeds the optimum level, microstructural heterogeneity becomes more pronounced due to particle agglomeration [58,59].

Another important observation is the apparent reduction in microvoids in EBC 2 and EBC 3 compared with EBC 1. The GNP appears to fill interstitial spaces between coconut shell particles and the epoxy matrix, resulting in a denser composite structure. This densification effect agrees well with the experimentally observed increase in bulk density from 1137.5 kg/m^3^ for EBC 1 to higher values for graphene-containing formulations. The enhanced compactness also contributes to improved thermal transport pathways and greater dielectric polarization efficiency.

The SEM results further support the thermal analysis findings. The more compact and interconnected morphology observed in graphene-containing composites provides additional barriers against heat transfer and volatile diffusion during thermal degradation. The improved thermal stability and higher residual char yields observed in TGA analyses can be partially attributed to the microstructural changes induced by graphene incorporation.

The SEM investigation confirms that GNP plays a critical role in modifying the microstructure of CSP-reinforced EBCs. Uniform graphene dispersion at low and moderate concentrations significantly improves interfacial adhesion, structural compactness, and crack resistance. Among the investigated formulations, EBC 3 exhibits the most homogeneous morphology, the strongest filler–matrix interaction, and the most efficient reinforcement mechanism, which explains its superior mechanical performance. In contrast, the more heterogeneous structure observed in EBC 4 suggests the onset of graphene agglomeration, highlighting the existence of an optimum graphene concentration for achieving balanced multifunctional properties in EBCs.

**Figure 12:**
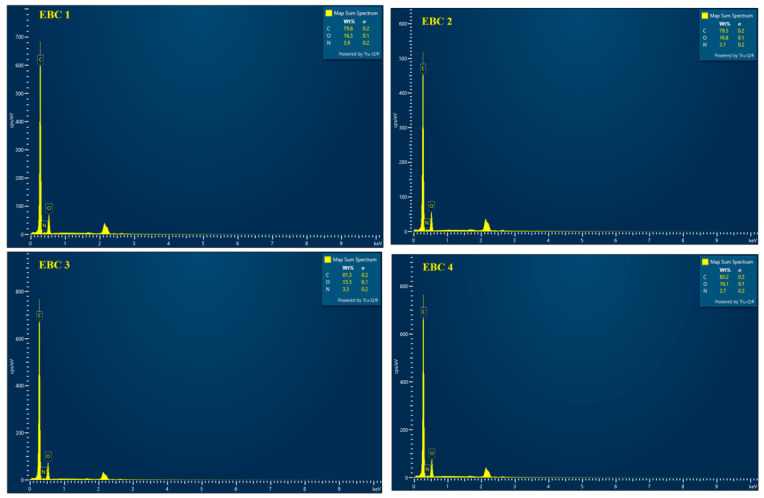
EDX results of EBCs.

Figure 12 presents the EDX spectra of the EBCs reinforced with CSP and different concentrations of GNP. The EDX analysis was performed to investigate the elemental composition of the composite surfaces and to verify the successful incorporation and distribution of the reinforcing phases within the epoxy matrix. The spectra of all samples exhibit similar elemental profiles, indicating that the fundamental chemical composition of the composites remained unchanged despite the addition of GNP. The dominant elements detected in all formulations were carbon and oxygen, which are characteristic constituents of both the epoxy matrix and lignocellulosic CSP.

The EDX spectra of EBC 1 is primarily dominated by a strong carbon peak accompanied by a smaller oxygen peak. The high carbon content originates from the aromatic and aliphatic carbon structures present in the cured epoxy resin as well as the cellulose, hemicellulose, and lignin components of the CSP. The oxygen signal is associated with hydroxyl, ether, carbonyl, and epoxy-derived oxygen-containing functional groups. The absence of additional significant elemental peaks indicates that the composite is free from major inorganic contaminants and confirms the purity of the raw materials used during fabrication [57,58,59].

For EBC 2, the EDX spectra exhibit a slight increase in the relative intensity of the carbon peak compared with EBC 1. This behavior is expected because GNP consists almost entirely of graphitic carbon. The increased carbon contribution provides indirect evidence of successful graphene incorporation into the epoxy matrix. The oxygen peak remains clearly visible, demonstrating that the addition of graphene does not alter the fundamental oxygen-containing structure of the epoxy–coconut shell system. The relatively smooth elemental distribution observed in conjunction with SEM analysis suggests homogeneous dispersion of graphene at this concentration.

The spectrums of EBC 3 show a further enhancement of carbon intensity while maintaining a similar oxygen signal. This observation is consistent with the increased graphene loading and supports the SEM findings indicating excellent graphene dispersion within the matrix. The balanced elemental distribution suggests strong interfacial compatibility between the GNP, coconut shell particles, and epoxy resin. The absence of unexpected elemental peaks further indicates that no secondary reaction products or contamination were introduced during composite preparation. The improved carbon-rich microstructure observed in EBC 3 is likely responsible for its superior tensile strength, hardness, thermal conductivity, and thermal stability.

In the case of EBC4, the carbon peak becomes slightly more pronounced owing to the higher graphene content. Although the overall elemental composition remains similar to that of the previous samples, the increased carbon concentration confirms the successful incorporation of additional GNP into the composite structure. The oxygen peak remains detectable, indicating that the epoxy matrix continues to constitute a substantial portion of the material. When interpreted together with the SEM observations, the EDX results suggest that graphene-rich domains may begin to form at higher graphene concentrations, although no evidence of chemical degradation or phase separation is detected.

A comparative evaluation of all EDX spectra demonstrates that carbon is the predominant element in every composite formulation, while oxygen constitutes the second major element. This finding is consistent with the chemical structures of epoxy resin, CSPs, and GNPs. The gradual increase in carbon peak intensity from EBC 1 to EBC 4 corresponds directly to the progressive increase in graphene loading. Such behavior confirms that GNP was successfully incorporated into the EBCs and remained present on the analyzed fracture surfaces.

The absence of additional elemental peaks is particularly important because it indicates that no undesirable impurities, catalyst residues, or inorganic contaminants were introduced during processing. Furthermore, the EDX results reveal that graphene incorporation mainly affects the quantitative elemental distribution rather than generating new chemical species. This observation is in excellent agreement with the FTIR analysis, where no new absorption bands were observed after graphene addition, suggesting that the reinforcement mechanism is predominantly physical rather than chemical.

The EDX findings also provide support for the thermal and mechanical performance enhancements observed in the graphene-containing composites. The increased carbon-rich structure contributes to improved thermal resistance through enhanced char formation during thermal degradation. Moreover, the highly conductive graphitic carbon network promotes more efficient stress transfer and thermal transport throughout the composite. The gradual increase in carbon content detected by EDX correlates well with the experimentally observed improvements in thermal conductivity, dielectric constant, tensile strength, and thermal stability.

The EDX analysis confirms the successful fabrication of graphene-reinforced EBCs and demonstrates that carbon and oxygen are the principal constituent elements of the materials. The progressive increase in carbon concentration with increasing graphene content, combined with the absence of foreign elemental impurities, verifies the effectiveness of the composite preparation procedure. When considered together with the SEM, FTIR, TGA, and mechanical characterization results, the EDX spectra provide strong evidence that GNP was uniformly incorporated into the epoxy matrix and contributed significantly to the enhanced multifunctional performance of the developed EBCs.

## 4. Conclusions

Sustainable EBCs reinforced with CSP and GNP were successfully fabricated, and their physical, mechanical, thermal, dielectric, and morphological properties were systematically investigated. The incorporation of GNP significantly enhanced the overall performance of the developed biocomposites. Bulk density, thermal conductivity, dielectric constant, and thermal stability increased progressively with increasing GNP content, while the tensile strength and Shore D hardness reached optimum values at 0.45 wt.% GNP. Specifically, the tensile strength increased by approximately 31%, demonstrating the effective reinforcing capability of uniformly dispersed GNPs and the improved interfacial interaction between the epoxy matrix and CSP. However, further increasing the GNP content to 0.75 wt.% resulted in a reduction in mechanical performance due to nanoparticle agglomeration and the formation of localized stress concentration sites.

TGA confirmed that graphene improved the thermal stability and residual char yield of the composites, whereas FTIR, SEM, and EDX analyses verified the successful incorporation of GNP without altering the fundamental chemical structure of the epoxy matrix. Among all formulations, EBC 3 containing 0.45 wt.% GNP exhibited the most balanced combination of tensile strength, hardness, thermal conductivity, dielectric behavior, thermal stability, and microstructural homogeneity, making it the optimum composition investigated in this study. The findings demonstrate that the synergistic combination of renewable CSP and GNPs provides an effective strategy for developing sustainable multifunctional EBCs with promising potential for lightweight structural and dielectric engineering applications. Future work will focus on comprehensive mechanical characterization, long-term durability, electrical performance, flame retardancy, and large-scale processing to further expand the application potential of these hybrid composites.

## Figures and Tables

**Table 1:** Composition of produced EBCs.

Experiment No.	Epoxy A (wt.%)	Epoxy B (%)	CSP (wt.%)	TEDA (wt.%)	Graphene (wt.%)
EBC 1	58.86	29.44	8.8	2.9	0.00
EBC 2	58.66	29.34	8.8	2.9	0.30
EBC 3	58.56	29.29	8.8	2.9	0.45
EBC 4	58.46	29.24	8.8	2.9	0.60
EBC 5	58.36	29.19	8.8	2.9	0.75

## Data Availability

The raw data supporting the conclusions of this article will be made available by the authors on request.

## Abbreviations

The following abbreviations are used in this manuscript:

ASTM	American Society for testing and materials
ATR	Attenuated total reflectance
CSP	Coconut shell powder
EBC	Epoxy-based biocomposite
EDX	Energy-dispersive X-ray spectroscopy
FTIR	Fourier transform infrared spectroscopy
GNP	Graphene nanopowder
SEM	Scanning electron microscopy
TEDA	Triethylenediamine
TGA	Thermogravimetric analysis
TLS	Thermal conductivity measurement system
